# Parameter design and effectiveness evaluation of wide strip mining of extra thick coal seams under dense buildings

**DOI:** 10.1038/s41598-024-60719-x

**Published:** 2024-05-02

**Authors:** Wulin Lei, Lan Yu, Shangyu Bai, Yanfeng Chen, Feng Zhao, Chao Zheng, Xu Huang, Jiangyi He, Hualong Zhou

**Affiliations:** 1 School of New Energy, Longdong University, Qingyang, 745000 Gansu China; 2 College of Energy Engineering, Xi’an University of Science and Technology, Xi’an, 710054 China; 3 Chenjiagou Coal Mine, Huating Coal Industry Group Co., Ltd., Pingliang, 744100 China; 4 Daliu Coal Mine, Huating Coal Industry Group Co., Ltd., Chongxin, 744000 China

**Keywords:** Three unders in coal mining, Wide strip mining, Probability integral method, Numerical simulation, Surface deformation prediction, Energy and society, Environmental impact

## Abstract

In order to safeguard the surface structures from mining damage while optimizing the liberation of coal resources under the dense surface buildings of the Cedi River coal mine. Considering that the analysis of the structure and type of surface buildings and the geological mining conditions of the mine, a wide strip mining design with a retention width of 70 m and a mining width of 50 m was finally determined by using the pressure arch theory and Wilson’s theory, combined with the actual layout of working faces 51,002, 51,004 and 51,006 at the site.The strip mining design is verified by probability integral method and FLAC^3D^ numerical simulation calculation respectively, The findings indicate that the highest value of earth surface subsidence created by the mining of the wide strip is 210 mm, the surface horizontal deformation value is 1.0 to − 1.4 mm/m, the damage to surface buildings is less than Level I, which satisfying the prerequisites of the surface building protection level, and can realize the continuous advancement of mine 51,002, 51,004 and 51,006 working faces, The coal pillars of the retained strip have sufficient support strength and long-term consistency, and the movement and deformation of the overburden after mining will not cause undulating subsidence of the surface, which effectively solve the mine's technical difficulties in safely coal mining under surface buildings.

## Introduction

The issue of coal mining beneath buildings is becoming more prevalent as China’s coal mining industry grows in size and intensity, strategies to optimize the liberation of coal mined beneath structures, rivers, and railroads, increase the amount of recoverable coal reserves, prolong the mine's useful life, and there is a genuine technical issue that needs to be resolved in order to fairly increase the rate of coal resource recovery^1^. According to the statistics on key coal mines in China, the amount of coal pressed under buildings, China's railway and water body weight is approximately 13.79 billion tons, of which the amount of coal pressed under buildings reaches about 8.76 billion tonnes^2^, however, the proportion of coal pressed by villages and towns among the coal pressed by buildings is over 60%. The technical difficulty of coal mining under buildings, the coal resources' poor pace of recovery, the large amount of mine development roadway works, and the relatively short service time of working face can very easily cause problems such as production succession tension^3,4^.

In the past 40 years, thanks to the technical research and mining practice of research institutes and production sites in the field of coal mining under villages and buildings, a lot of actual measurement data and successful experience have been obtained, making the coal mining technology under villages and buildings in China increasingly sophisticated. Zhou describes technical measures to resist the structural system of deformation buildings, the ability to resist the effects of mining and the economic benefits^5,6^. Li analysed the pressure distribution law within the coal pillar when the coal under the building is pressed by the strip method from the different mining status of the quarry^7^. He and Huang developed a sensitivity discriminant approach and a mechanical model for the anti-slip stability of coal pillars in strip mining ^8,9^. Zhu proposed a method to control surface movement and deformation by arranging extremely inadequate working faces according to local conditions^10^. Tan proposed that beam bending theory could be used to study buildings in mining area^11^. Hao was the first to successfully develop a comprehensive mechanised coal mining technology under buildings in the XingTai mine^12^. Guo proposed a design method for calculating the size of coal pillars for wide strip mining in deep wells for the characteristics of wide strip mining in deep wells^13^. Yu looked at strip mining's application to the law of surface movement and deformation beneath heavy layers of loess^14^. Li calculated the changes in the stress field, the working face's plastic zone and displacement field under the strip mining technique using numerical simulation^15,16^. Kuai projected the surface movement for coal mining under the industrial plaza at a mine in the Handan mining area, assessed the damage levels of major buildings^17^. Yu investigated the progressive stripping behaviour of strip mining coal pillars occurring under the action of various factors such as groundwater, established a model for non-uniform stripping of coal pillars, and finally gave an evaluation method for the long-term stability of coal pillars^18^. Guo explored a safe mining solution for working faces with small wide strips under dense buildings in industrial plazas^19^. Song proposed a four-stage strip paste filling mining technique under buildings and determined reasonable process parameter^20^. Gao used replacement strip coal pillar mining, a "mining-retaining-filling" combination of loss reduction mining method^21^. Most of the available research has been conducted on surface movement and deformation in conventional strip mining, while less research has been conducted on wide strip mining under buildings, moreover, has focused mainly on the analysis of overburden as well as surface movement and deformation, while little analysis has been done to summarise the basic calculation of coal pillar retention parameters and stability.

This article aimed to achieve safe coal mining under the buildings of Cedi River coal mine, by using various methods such as field survey, theoretical calculation of strip mining parameters and FLAC^3D^ numerical simulationthe geological mining conditions and the condition of the surface buildings are analysed on the basis of maximising the extraction of coal resources pressing under the buildings and protecting the safety of the surface buildings, and a design scheme of wide strip mining was proposed, which ensures the safe use of the surface buildings and maximises the recovery rate of coal resources, and is of great significance to achieve green, efficient and safe mining in the mine.

## Engineering background

### Geological mining conditions

Cedi River coal mine is located in the northern part of the Huating coalfield. The mineral rights extend from the F1 Tangjiashan reverse fault in the north, with the technical boundary to the south and west, the western oblique structure and the F_2_ reverse fault, and to the south of the coal seam outcrop line in the east. The mine has a design capacity of 0.9 Mt/a, a strike length of 4.5 km, an inclined length of 2.5 km, a field area of 7.50 km^2^ and a mining depth of 350–440 m. The northern part of the field is buried shallowly and the southern part deepeer. The research area is characterised by low hilly terrain with sporadic outcrops of local bedrock. The main recoverable coal seam is 5# coal, with an average thickness of approximately 8.0 m and a dip angle of 22°–37°. The coal seam is located in the northeast of the wellfield at a relatively large dip angle, and in the southwest at a relatively small dip angle. The coal seam is stable and generally mineable within the wellfield. The area is a complex diagonal structure with inclined coal seams and simple hydrogeological conditions. The overlying rocks in the study area are mainly mudstones, siltstones, medium-grained sandstones as well as coarse and fine sandstones, the overlying rocks are of medium to hard lithology. The rock parameters are shown in Table 1.

**Table 1:** Characteristic table of roof and floor of coal seam.

No.	Rock layer name	Strata	Thickness (m)	Elastic modulus (GPa)	Compressive strength (MPa)	Bulk density (g/cm^3^)
1	Main roof	Siltstone	14.5	1.8	21.3	2.5
2	Immediate roof	Fine sandstone	3.5	1.4	18.0	2.4
3	5# coal	Coal	8.0	1.3	16.0	1.4
4	Immediate floor	Mudstone	3.0	1.5	15.0	2.5

### Overview of surface buildings

**Figure 1:**
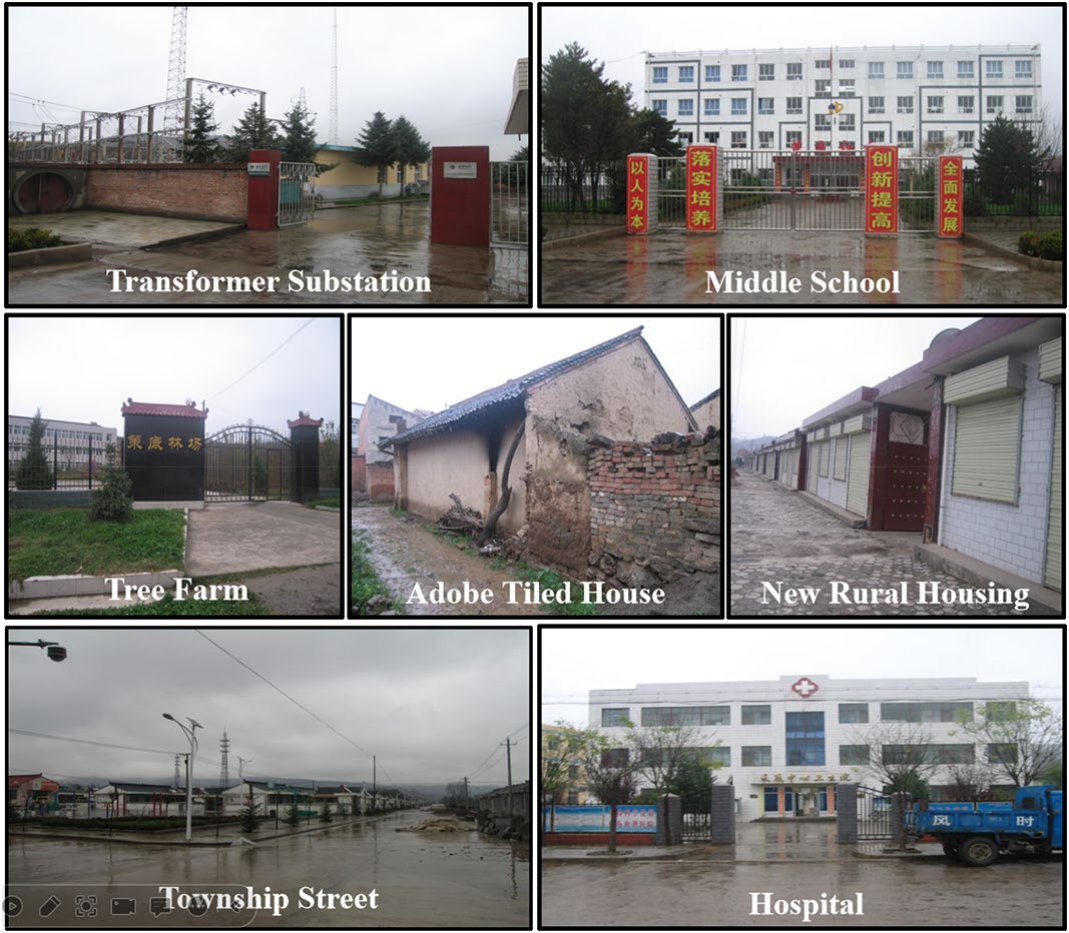
Representative buildings on the surface section.

The ground level of the Cedi River coal mine involves a high density, large number, complex structure and wide distribution of protected buildings. The ground level buildings consist of 1 to 3-storey buildings and bungalows, etc. Some of the buildings are shown in Fig. 1. The coal pressing under the buildings of Cedi River Coal Mine is distributed in the central area along the strike of 51,002, 51,004 and 51,006 faces in the southern flank of the area, with a pressed coal width of approximately 600 m. It is initially estimated that the pressing coal under the buildings of Cedi River Coal Mine accounts for approximately 3.69 Mt of the 5# coal reserves, accounting for 19% of the 5# coal recoverable reserves.

The buildings on the surface of the Cedi River coal mine can be classified by type of structure: 464 brick and wood structured brick cottages, 178 brick and concreate structured brick buildings, 74 brick and soil structured adobe cottages, and 2 steel frame structured steel sheds. They can be classified according to their use: residential houses, commercial buildings, office buildings, schools, churches, town public facilities, etc. According to the Regulations for the Retention of Buildings, Water Bodies, Railways and Coal Pillars in Major Shafts and Pressed Coal Mining^22^, the protection level of surface buildings is divided as shown in Table 2.

**Table 2:** Building protection classification.

Building name	Types	Structure	Quantity	Level
Transformer substation	Bungalow	Brick and wood structure	1	II
Middle school, Hospital	Building	Brick and concrete structure	33	II
Second floor residential buildings, Shops	Building	Brick and concrete structure	178	II
First floor residential buildings, Shops	Bungalow	Brick and tile structure	464	III
First floor residential buildings, Shops	Bungalow	Brick and wood structure	74	III

The protection level of the main buildings (structures) on the surface of the Cedi River coal mine is II–III. In view of the following factors, the overall protection level of the surface buildings (structures) is determined to be II.

(1) Integrated consideration of underground public utilities such as street water supply and electricity supply; (2) Surface II and III protected buildings overlap and cross each other; (3) The design and construction of buildings (structures) are not sufficiently standardized, and some buildings are not provided with deformation joints in accordance with the regulations; (4) Some brick and earth and brick houses are in a poor state of repair and have poor resistance to deformation, and may suffer damage when the surface deformation exceeds Grade I.

## Wide strip mining scheme design

### Strip mining overview

**Figure 2:**
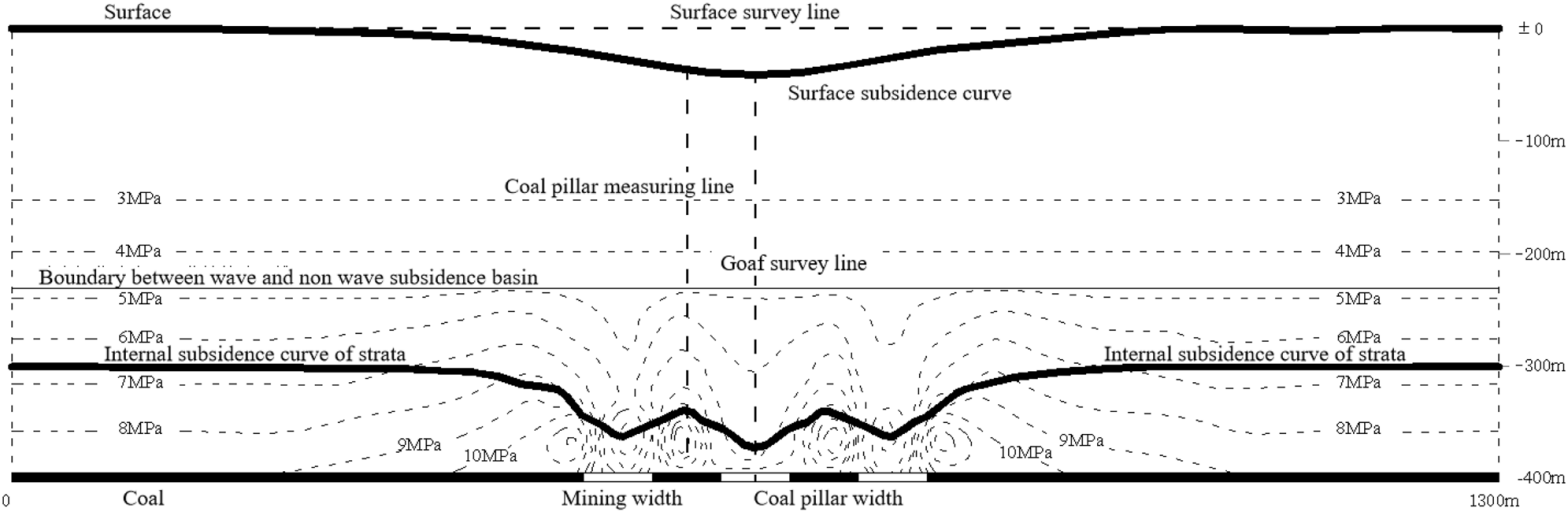
Surface subsidence mechanism of strip mining.

Strip mining technology^23^ is a method of partially mining resources, the essence of which is to divide the coal seams in the mining area into relatively regular strips, mining one, retaining one, and making the retained coal pillars have enough deformation to support the overburden, with only relatively small movement of the surface, thus not affecting the surface structures. its mining surface subsidence mechanism is shown in Fig. 2.

Strip mining should ensure that the coal pillars left in place have sufficient strength and long-term stability, while ensuring that the surface deformation caused by mining meets the requirements of the protected object. Field practice shows that strip mining should meet the following principles^24^.

**Figure 3:**
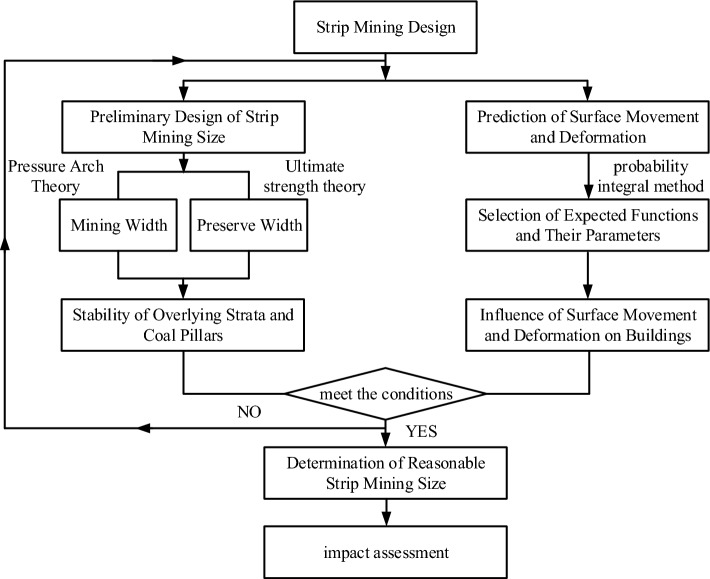
Strip mining design procedure flow chart.

(1) Permissible mobile deformation of the ground surface. After strip mining, the surface forms a uniform single subsidence basin without wave-like subsidence; the value of mobile deformation generated by the surface should be much smaller than the value of deformation allowed for surface buildings; (2) Coal pillar stability. The strip coal pillars left in place are of sufficient strength to support the overburden loads and to maintain their long-term stability; (3) Recovery rate. The recovery rate should be as large as possible and the strip extracted as wide as possible, provided that the principles of permissible surface deformation; (4) The width to height ratio of the retained strip should be not less than 2 (infill mining) or not less than 5 (collapse mining); (5) Generally speaking, the strip should be mined at a width of no more than 1/4 of the mined seam's buried depth; (6) Strip mining and retention width arrangement should be adopted according to the characteristics of surface construction distribution in order to reduce the surface deformation damage caused by mining. The design flow of which can be described in Fig. 3.

### Design of mining width b

According to domestic and international strip mining experience, in order to ensure that the surface does not sink in waves, but rather in a single gently sinking basin, the mining width b leaving^25^ should follow the following principles:

1 $$b = \left( {\frac{1}{4} \sim \frac{1}{10}} \right)H$$

In the formula, *H* is the mining depth, m; *b* is the mining width, m.

The main mining 5# coal seam is buried at a depth of approximately 350–440 m. The minimum mining depth value is used to determine the mining width range. Based on the above formula, the mining width is calculated to be between 35.0 and 87.5 m.

As the pressure arch is formed in the upper part of the mining area according to the pressure arch theory, only part of the load of the overburden rock acts on the direct roof slab and most of it acts in the solid coal arch foot area in the face. Therefore, the formula for calculating the internal width *L*_*PA*_ of the pressure arch is as follows:

2 $$L_{PA} = 3 \times \left( {\frac{H}{20} + 6.1} \right)$$

If the "partial strip mining" method is used to control surface subsidence, the strip mining width should be left to meet the following principles:

3 $$b \le 0.75L_{PA}$$

Based on the above formula, the mining width should be less than 53.1 m.

The old roof of the coal seam continues to develop upwards in the form of a beam to control the rock fall damage of the coal seam roof, forming a control structure to support the role of the overburden, making the surface movement deformation is small and gentle, the total amount of surface movement is about the sum of the coal column, overburden compression and old roof deflection. The mining width was finally determined to be 55 m.

### Design of mining width a

Based on Wilson’s three-way stress method coal pillar design theory^26^, the size of retained coal pillars for strip mining generally needs to satisfy the following relationship:

4 $$a > 2Y + S = 0.01MH + S$$

In the formula, *Y* is the coal pillar yield width, m; *S* is the coal pillar nucleation zone width, m; *M* is the coal seam thickness, m.

Bringing *S* = 8.4 m, *M* = 8.0 m and *H* = 440 m into the equation gives a stay width *a* > 43.6 m.

According to experience, the width of the coal pillar should be greater than 0.12 *H*, thus *a* > 52.8 m. Considering that there are buildings with a higher level of protection such as substations and schools on the surface. The width of the coal pillar should be increased appropriately, given that the return roadway of the 51,002 face has been formed and the length along the inclination is 130 m, the width of the retained coal pillar is determined to be 75 m.

### Coal pillar stability verification

Using Cullen's criterion and Wilson's theory, the ultimate load carrying capacity of the coal pillar is verified as a factor of safety *k*, calculated for long coal pillars^27^, and the ultimate load carrying capacity of the coal pillar is:

5 $$F_{{\text{c}}} = 40h\gamma (a - 4.92mh \times 10^{ - 3} )$$

When the width of goaf on both sides is less than 0.6 *H*, the actual load sharing capacity of the coal pillar is:

6 $$F_{{\text{d}}} = 10\gamma [ha + {{\text{b}} \mathord{\left/ {\vphantom {{\text{b}} 2}} \right. \kern-0pt} 2}(2h - {{\text{b}} \mathord{\left/ {\vphantom {{\text{b}} {0.6}}} \right. \kern-0pt} {0.6}})]$$

The coal pillar stability factor of safety *k* is then:

7 $$k = {{F_{{\text{c}}} } \mathord{\left/ {\vphantom {{F_{{\text{c}}} } {F_{{\text{d}}} }}} \right. \kern-0pt} {F_{{\text{d}}} }}$$

In the formula, *F*_*c*_ is the ultimate load carrying capacity of coal pillar, kN; *F*_*d*_ is the load sharing capacity of coal pillar, kN; *k* is the safety factor of coal pillar stability; *h* is the thickness of overburden rock, m; *γ* is the average overburden rock capacity, kN/m^3^; *m* is the height of coal pillar, m; *a* is the width of coal pillar, m; *b* is the mining width, m.

The calculations show: *k* = 1.85 ≥ 1.5. Therefore the technical requirements for the safety factor for collapse strip mining are met.

Coal pillar aspect ratio and height to width ratio checks:

Length/width = 8 > 3, width/height = 9.38 > 5. The coal pillar designs all meet the technical requirements for strip mining pillars.

Calculation of coal pillar yield zone width:

8 $$r_{p} = \frac{hd}{{2tg\phi }}\left\{ {\ln \left[ {\frac{{C + \sigma_{zl} tg\phi }}{{C + \frac{{P_{x} }}{\beta }tg\phi }}} \right]^{\beta } + tg^{2} \phi } \right\}$$

In the formula, *r*_*p*_ is the width of the yield zone of the coal pillar, m; *h* is the height of the coal pillar, m; *d* is the factor of mining disturbance, d = 1.5–3.0; *β* is the lateral pressure coefficient at the interface between the yield zone and the nucleus; *C* is the cohesive force at the contact surface of the coal seam and the top and bottom plates, MPa; *ϕ* is the friction angle at the contact surface of the coal seam and the top and bottom plates, °; *σ*_*zl*_ is the strength at the limit of the coal pillar, MPa; *P*_*x*_ is the lateral constraint force of the coal wall, MPa.

Based on the geological data of the Cedi River Mine and experience, the following parameters were derived:

Substituting *d* = 2.0, *β* = 0.3, *C* = 0.8 MPa, *φ* = 25°, *σ*_*zl*_ = 8 MPa and *P*_*x*_ = 1 Mpa into Eq. (8) gives *r*_*p*_ = 7.54365 m.

Coal pillar nucleation zone ratio check:

9 $$R = \left( {a - 2r_{p} } \right)/a$$

In the formula, *R* is the coal pillar nucleation zone ratio, %.

According to the mechanics of the rock, the yield zone in the steady state of the coal column must not only satisfy the stress balance equation, but must also satisfy the strength criterion. The formula for calculating the width of the yield zone of a coal pillar is derived from the Coulomb criterion and the calculated kernel curvature of a strip coal pillar is greater than 65%. The calculated nucleation zone rate *ρ* = 0.7990.65 for a stable coal pillar meets the nucleation zone rate requirement for a strip stay wide coal pillar^28^.

Strip recovery rate calculation:

10 $$K = b/(a + b)$$

In the formula, *K* is the recovery rate, %.

Bringing the mining width of 55 m and leaving width of 75 m into Eq. (10), the strip mining area recovery rate is calculated to be 42.3%, which meets the technical requirements.

In summary, the comprehensive parameters of coal pillar stability are shown in Table 3, and it was finally determined that the strip mining design with mining width *b* = 55 m and leaving width a = 75 m meets the requirements and that the design has sufficient strength and long-term stability.

**Table 3:** Comprehensive calculation parameter table for stability of coal pillar.

Coal	Mining depth (m)	Mining height (m)	Reserved width (m)	Mining width (m)	Safety factor	Width of coal pillar yield zone (m)	Ratio of width to height (%)	Core area ratio of coal pillar (%)	Recovery rate (%)
5#	440.00	8.00	75.00	55.00	1.85	7.54	9.38	79.90	42.30

## Surface movement deformation predicted

### Probabilistic integral method projection model

A large number of observations have shown that the degree of damage in the area affected by mining is very much related to the rate of subsidence of the surface, but the rate of subsidence is also related to the mining time, mining depth, overburden lithology and other factors of each mining block. Therefore, the mathematical model of surface movement and deformation must include the above-mentioned influence factors, and the universal model is in line with the above characteristics. The expression of the mathematical model is as follows:

11 $$D(x,y,z) = \sum\limits_{i = 1}^{m} {C_{i} } \sum\limits_{k = 1}^{l} {\int\limits_{{q_{k} }}^{{q_{k + 1} }} {f_{j1} (R_{k} ,z)f_{j2} (q)dq} }$$

In the formula, *(x, y, z)* is the coordinates of the calculation point; *C*_*i*_ is the time influence coefficient; *m* is the number of calculated block segments; *l* is the number of inflection points of any of the calculated mining segments; *f*_*j1*_*, f*_*j2*_ is the operation function.

Radius of polar coordinates:

12 $$Rk = Rk(q,x,y) = \frac{{(x_{k} - x)(y_{k + 1} - y_{k} ) - (y_{k} - y)(x_{k + 1} - x_{k} )}}{{(y_{k + 1} - y_{k} )\cos q - (x_{k + 1} - x_{k} )\sin q}}$$

In the formula, *x*_*k*_*, y*_*k*_ are the coordinates for calculating the inflection point of the mining block, where *x*_*k*+*1*_ = *x*_*1*_ and *y*_*k*+*1*_ = *y*_*1*_ when *k* ≥ *m*. The above formulae are the universal model applied in the mining damage prediction evaluation system.

**Figure 4:**
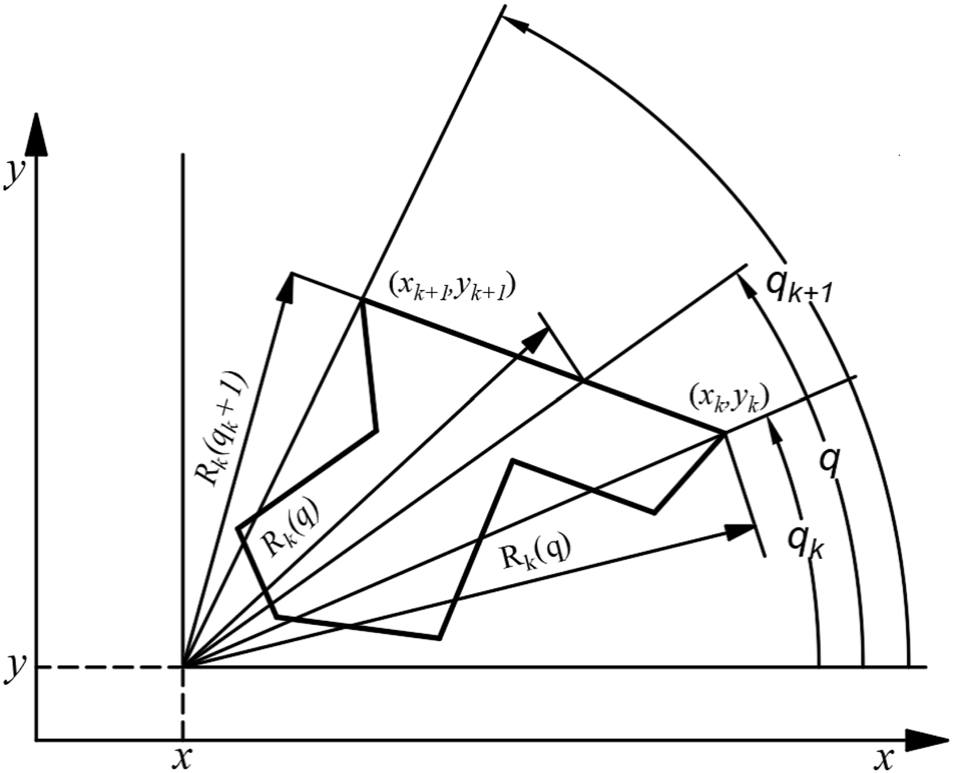
Schematic diagram of the principle of integration in polar coordinates.

Using the probabilistic integration method as a basis, the principle of polar integration is illustrated in Fig. 4, and the expected formula for the sink value of the mined area in the above mathematical model is:

13 $$w(x,y,t) = \frac{{w_{\max } }}{2\pi }C_{i} \cdot \sum\limits_{k = 1}^{l} {\int\limits_{{q_{k} }}^{{q_{k + 1} }} {\left[ {1 - e^{{ - \pi \frac{{R_{k}^{2} }}{{r^{2} }}}} } \right]} dq}$$

In the formula, *r* = *H/tgβ* is the radius of major influence; *ω*_*max*_ is the maximum sinkage value for full surface mining; *C*_*i*_ = *(1 − e*^*cti*^*)* is the time factor for surface sinkage determined by mining depth, overburden lithology, and mining rate.

The model introduces a time factor in order to be able to predict more accurately the value of surface subsidence at different time periods of mining. In the equation *C*_*i*_ = *(1 − e*^*cti*^*)*, *c* is a factor given according to the depth of extraction and lithology. *t*_*i*_ is defined by the year and month *t*_*is*_ of the start of the mining block section and the year and month *t*_*ie*_ of the calculation time (*t*_*i*_ = *t*_*ie*_* − t*_*is*_). For the value of *c*, when no actual observations are available, if the mining rate is less than 5 m/day, For smaller mining depths (*H* < 500 m), and softer overburden lithology, 3.0 ≥ *c* ≥ 2.5; for smaller mining depths (*H* < 500 m),and harder overburden lithology, 2.5 ≥ *c* ≥ 2.0; for larger mining depths (*H* > 500 m), and softer overburden lithology, 2.0 ≥ *c* ≥ 1.5; for larger general mining depths (*H* > 500 m), and harder overburden lithology, 1.5 ≥ *c* ≥ 1.0.

The above mathematical model is established for horizontal and near-horizontal coal seam mining. Due to the superimposability of mining subsidence, the following division principles should be followed when inclined coal seam mining is expected: the burial depth, mining height, dip angle, orientation, mining method, mining time, time of initial mining and re-mining, surface elevation, coal seam floor elevation and other mining geological conditions of the same calculation block are basically the same. This is one of the advantages of applying the mathematical model to this system, so that the mathematical model is applicable to both horizontal and near-horizontal coal seam mining, as well as to inclined coal seam mining.

In terms of the interrelationships between the ground movement deformation, the equations for each component of the tilt (*i*_*x*_*, i*_*y*_) and horizontal deformation *(K*_*x*_*, K*_*y*_), horizontal movement (*μ*_*x*_*, μ*_y_) and horizontal deformation (*ε*_*x*_*, ε*_*y*_) due to the expected dynamic subsidence of the ground surface can be derived from Eq. (13) as follows:

14 $$i_{x} = \frac{{w_{\max } }}{r}\sum\limits_{i = 1}^{m} {C_{i} \sum\limits_{k = 1}^{l} {\int\limits_{{q_{k} }}^{{q_{k + 1} }} {F_{1} \left( {\frac{{R_{k} }}{r}} \right)} } } \cos qdq$$

15 $$i_{y} = \frac{{w_{\max } }}{r}\sum\limits_{i = 1}^{m} {C_{i} \sum\limits_{k = 1}^{l} {\int\limits_{{q_{k} }}^{{q_{k + 1} }} {F_{1} \left( {\frac{{R_{k} }}{r}} \right)} } } \sin qdq$$

16 $$K_{x} = \frac{{w_{\max } }}{{r^{2} }}\sum\limits_{i = 1}^{m} {C_{i} \sum\limits_{k = 1}^{l} {(P_{k} - Q_{k} )} }$$

17 $$K_{y} = \frac{{w_{\max } }}{{r^{2} }}\sum\limits_{i = 1}^{m} {C_{i} \sum\limits_{k = 1}^{l} {(P_{k} + Q_{k} )} }$$

18 $$u_{x} = w_{\max } \sum\limits_{i = 1}^{m} {b_{i} C_{i} \sum\limits_{k = 1}^{l} {\int\limits_{{q_{k} }}^{{q_{k + 1} }} {} F_{1} \left( {\frac{{R_{k} }}{r}} \right)} } \cos qdq$$

19 $$u_{y} = w_{\max } \sum\limits_{i = 1}^{m} {b_{i} C_{i} \sum\limits_{k = 1}^{l} {\int\limits_{{q_{k} }}^{{q_{k + 1} }} {} F_{1} \left( {\frac{{R_{k} }}{r}} \right)} } \sin qdq$$

20 $$\varepsilon_{x} = \frac{{w_{\max } }}{{r^{2} }}\sum\limits_{i = 1}^{m} {C_{i} b_{i} \sum\limits_{k = 1}^{l} {(P_{k} - Q_{k} )} }$$

21 $$\varepsilon_{y} = \frac{{w_{\max } }}{{r^{2} }}\sum\limits_{i = 1}^{m} {C_{i} b_{i} \sum\limits_{k = 1}^{l} {(P_{k} + Q_{k} )} }$$

where $$F_{1} (s) = se^{{ - \pi s^{2} }} - \int\limits_{0}^{s} {e^{{ - \pi t^{2} }} dt}$$; $$F_{2} (s) = \pi s^{2} e^{{ - \pi s^{2} }}$$; $$F_{3} (s) = 1 - e^{{ - \pi s^{2} }} - \pi s^{2} e^{{ - \pi s^{2} }}$$; $$P_{k} = \int\limits_{{q_{k} }}^{{q_{k + 1} }} {F_{2} \left( {\frac{{R_{k} }}{r}} \right)} dq$$; $$Q_{k} = \int\limits_{{q_{k} }}^{{q_{k + 1} }} {F_{3} \left( {\frac{{R_{k} }}{r}} \right)\cos 2q} dq$$.

### Determination of expected parameters

The main factors influencing the expected parameters of mining subsidence are: lithology and stratigraphic structure of the overburden, mining depth and thickness. Currently, the predicted parameters for mining subsidence are usually determined by analogyon the basis of surface subsidence monitoring. An analogous method was used to determine the surface subsidence coefficient *η*_*f*_ = 0.7, at full mining, the angle tangent *tgβ*_*f*_ = 1.96 (*β* = 63°), at the main influence range, the inflection point offset distance *d*_*f*_ = 0.05 H, and the horizontal movement coefficient *b*_*f*_ = 0.3.

The probability integral method predicts that the correlation between parametric strip mining and normal full mining can be converted by the following equation:

22 $${\text{Subsidence factor}}:\;\frac{{\eta_{s} }}{{\eta_{f} }} = \frac{H - 30}{{5000{a \mathord{\left/ {\vphantom {a b}} \right. \kern-0pt} b} - 2000}}$$

23 $${\text{Inflection point offset distances}}:\;d_{s} = \frac{{1.56{\text{w}}_{{\text{e}}} H}}{{{\text{w}}_{{\text{p}}} \left( {0.01H + 30} \right)}}$$

24 $${\text{Horizontal movement factor}}:\;{\text{b}}_{s} = \left( {1.29 - 0.0026H} \right) \cdot {\text{b}}_{f}$$

25 $${\text{Major influences on angular tangency}}:\;{\text{tg}}\beta_{s} = \left( {1.076 - 0.0014H} \right) \cdot {\text{tg}}\beta_{f}$$

Formula: footnote to symbols, full-longwall full mining; strip-strip mining.

According to the empirical formula (22–25), when mining width is 55 m and leaving width is 75 m, the strip mining surface subsidence coefficient *η*_*s*_ is *0.04*, the main influence range angle tangent *tgβs* is *1.23*, the inflection point offset distance* d*_*s*_ is *0.55H.* The basic parameters of maximum surface deformation are shown in Table 4.

**Table 4:** Maximum moving deformation value of the surface.

Subsidence (mm)	Horizontal displacement (mm)	Horizontal deformation (mm/m)	Curvature (mm/m^2^)	Tilt (mm/m)
210	85	1.0 to − 1.4	− 8 to 20 × 10^−6^	1.8

**Figure 5:**
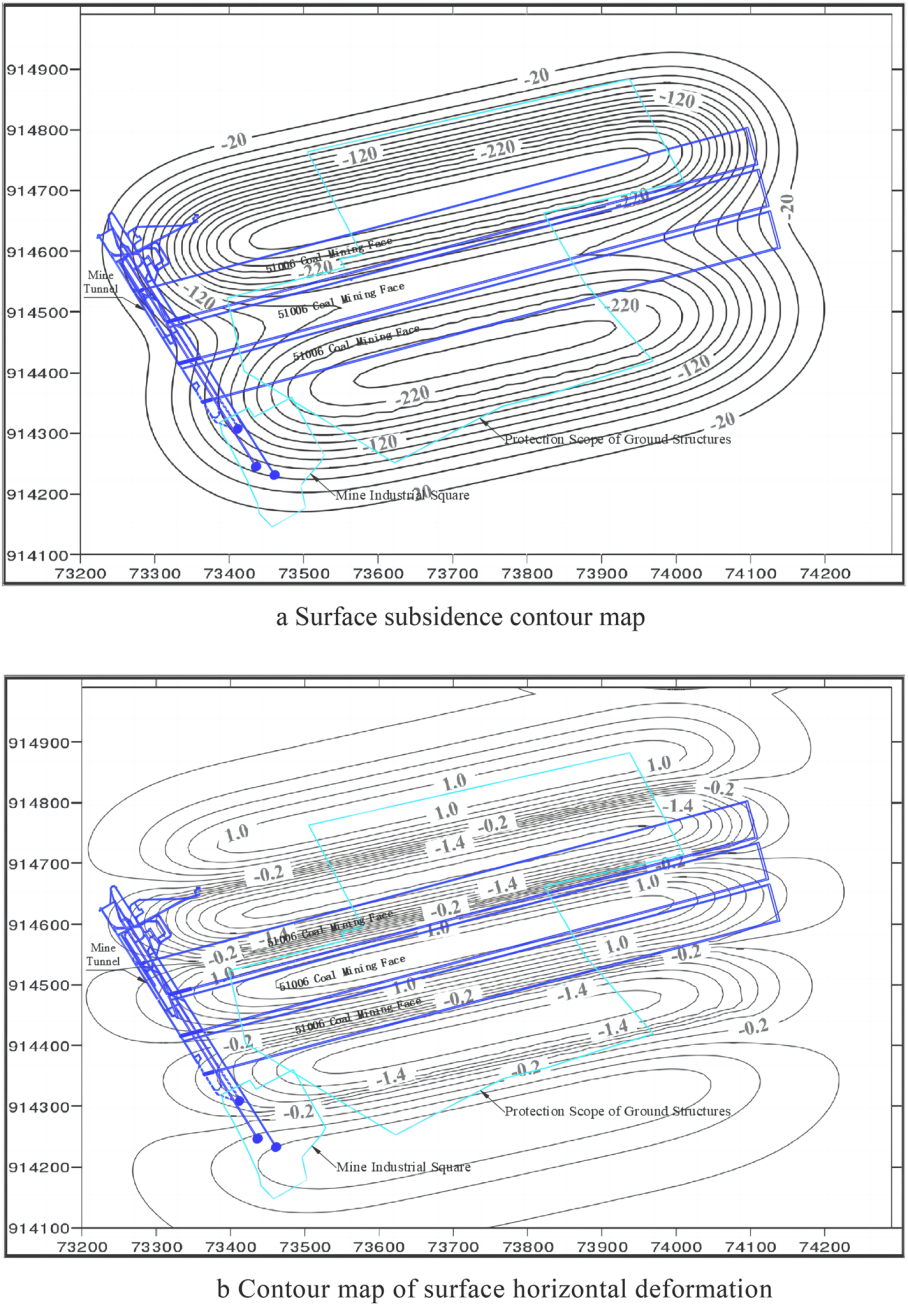
Contour analysis of surface movement and deformation after strip mining. (**a**) Surface subsidence contour map. (**b**) Contour map of surface horizontal deformation.

Based on the distribution of the designed protected coal pillars under the Cedihe coal mine buildings, the mining conditions of the 51,002, 51,004 and 51,006 working faces were analysed, the expected block sections were determined, the Ylh-12 probabilistic integral estimation program^29^ was applied to the calculations and processed as full basin moving deformation contours for analysing the feasibility of the mining scheme, and the results were slightly larger than the probabilistic integral method, as shown in Fig. 5.

The goal of strip mining is to safeguard the structures on both sides of the village center, per the design specifications for coal mining beneath the buildings of the Cedihe coal mine, while the rest of the buildings are designed for mining according to relocation or post-mining restoration. Based on a comprehensive analysis, and in compliance with the terms of the "Regulations for the Laying of Coal Pillars and Coal Pressing in Buildings, Water Bodies, Railways and Major Shafts", the main masonry buildings to be protected on the surface should be affected by mining to the extent of Class I. Only a small number of three-storey buildings are damaged to the extent of Class II, so strip mining will have less impact on the buildings. Therefore, this wide strip mining design scheme not only meets the technical requirements of mining, but also realises the principle of surface building protection.

## Numerical simulation analysis

### Model building

**Figure 6:**
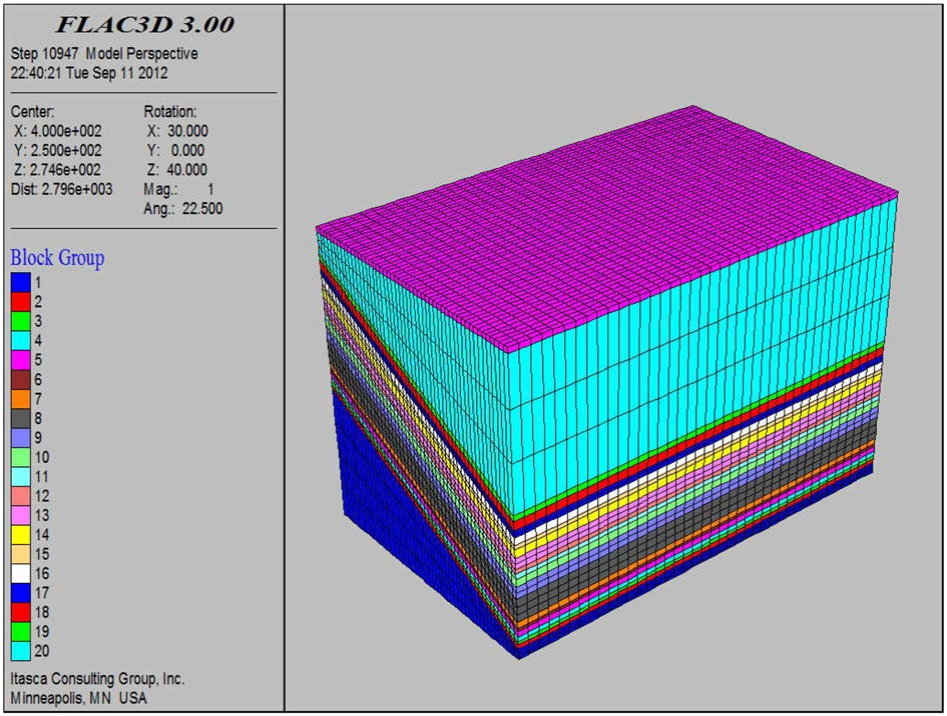
Three dimensional calculation model.

According to the design plan of wide strip mining under the building of Cedi River coal mine, FLAC^3D^ software^30^ was applied to simulate the mining of 51,002, 51,004, and 51,006 working faces, and the simulated working face had a tendency length of 120 m under the non-building, a mining width of 55 m under the building, a coal column width of 75 m, a strike advance length of 600 m, a coal thickness of 8 m, and a mining depth of 350–400 m. The model is excavated step by step from the position of the cutting eye, with each step excavating 5 m. After the model is calculated to be stable, the deformation, failure, and stress changes of the coal rock mass on the top and bottom of the mining face are recorded and analyzed before proceeding to the next step of development. A total of 600 m of coal seam is excavated. The final model size is 800 × 500 × 549 m, with 21 layers in total, and the model is divided into 62,000 meshes and 69,003 nodes; the Moore-Cullen strength criterion is selected, and displacement boundary conditions are used, based on geotechnical theory^31^, which can basically simulate the geological conditions of the site realistically in Fig. 6.

### Simulation results and analysis

**Figure 7:**
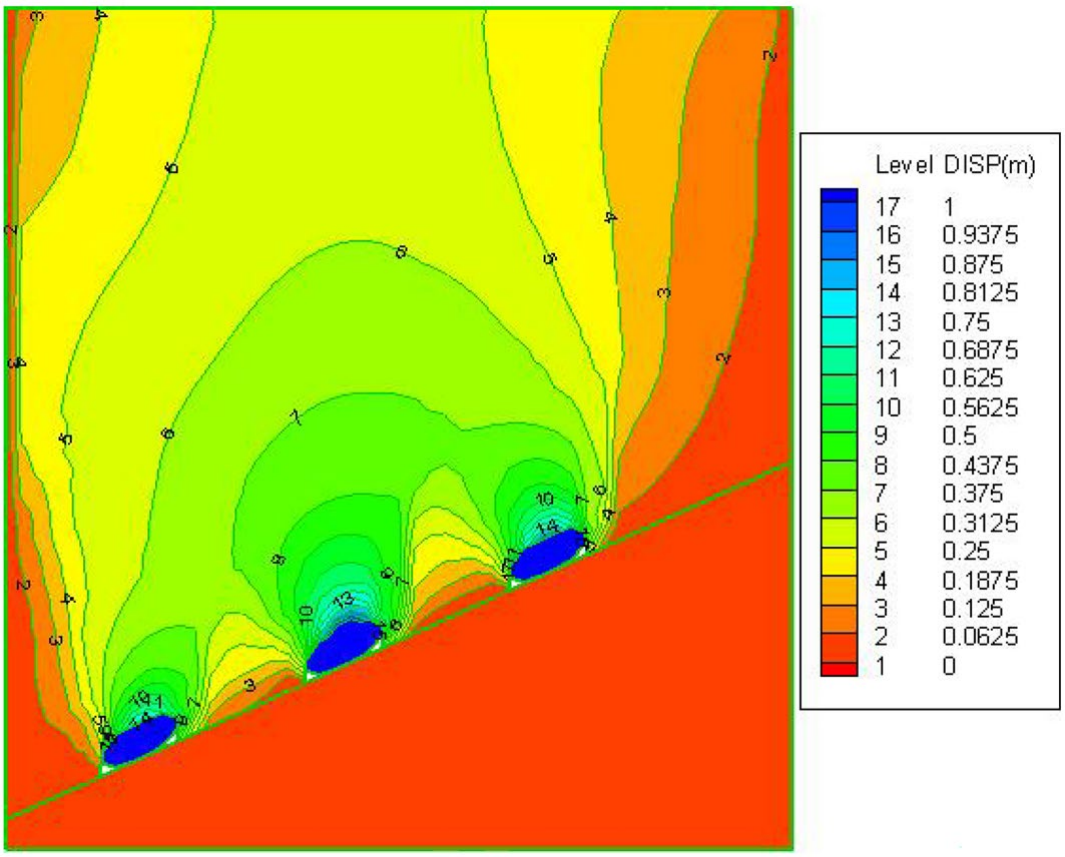
Cloud map of the vertical displacement.

Fafter FLAC^3D^ simulates the mining of three strips, the overburden movement deformation along the coal seam inclination direction profile is shown in Fig. 7, from the edge to centre of the coal pillar, the compression of the coal pillar gradually decreases, and near the centre of the coal pillar the compression of the coal pillar reaches the minimum value of this coal pillar compression, indicating that the middle is the nucleation zone, and the nucleation zone is in a three-way stress state, which effectively supports the roof and controls the movement of the overburden. From the analysis of the sinking law of the mining area and the overburden above the coal pillar, Immediately above the mining strip, the overburden's vertical distortion may be seen to be gradually decaying from botto.

**Figure 8:**
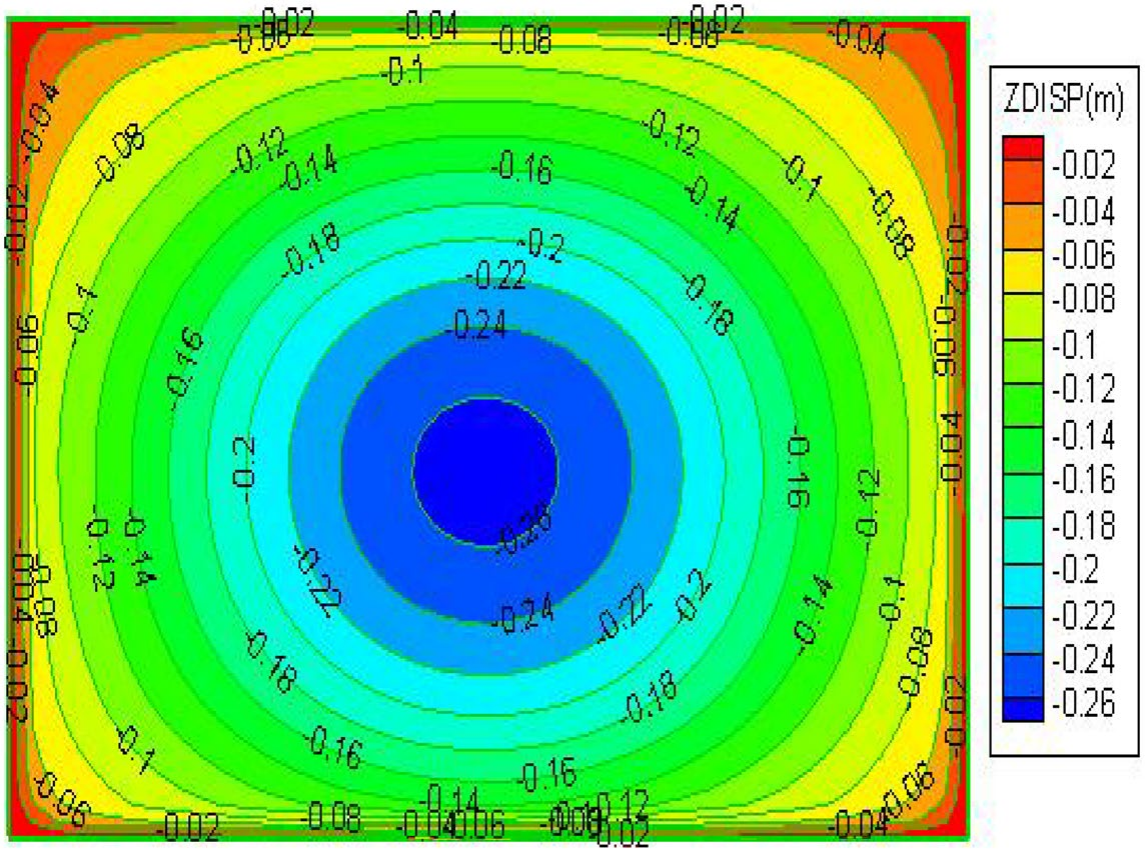
Post-mining surface subsidence cloud.

From the Fig. 8, Observing that the mining area's maximum surface subsidence value is situated in the middle of the surface, somewhat downward, suggests that the surface subsidence basin is skewed downward; and the contour lines in the downhill direction are denser than in the uphill direction, indicating that the surface deforms more in the downhill direction; the overall amount of surface subsidence is small and no wave subsidence occurs, with a maximum subsidence value of 266 mm.

**Figure 9:**
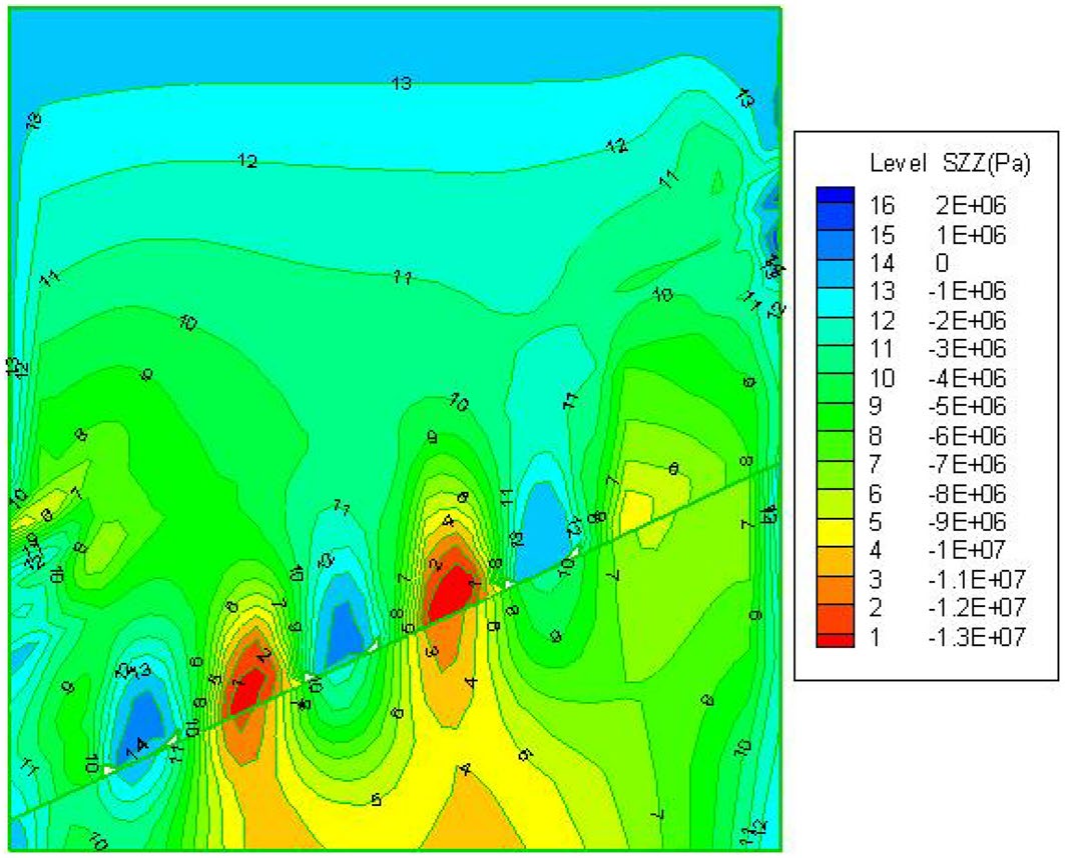
Cloud map of vertical stress distribution in inclined main section.

Figure 9 illustrates that a stress-balanced arch that approximates a semi-ellipse forms above each of the three mining zones. The overburden rock disturbance caused by coal mining gradually lessens vertically within the stress-balanced arch, relieving vertical tension and causing a commensurate decrease in ground surface movement and deformation; the stress value of the strip coal pillar gradually increases from the edge to the centre, with a maximum stress value of 13 MPa, indicating that the middle part of the coal pillar exhibits higher strength than the edge part of the pillar, which is mainly due to the lateral limiting effect of the edge of the pillar on the centre of the pillar, leaving the middle pillar in a three-way stress state, thus increasing the strength of the media.

**Figure 10:**
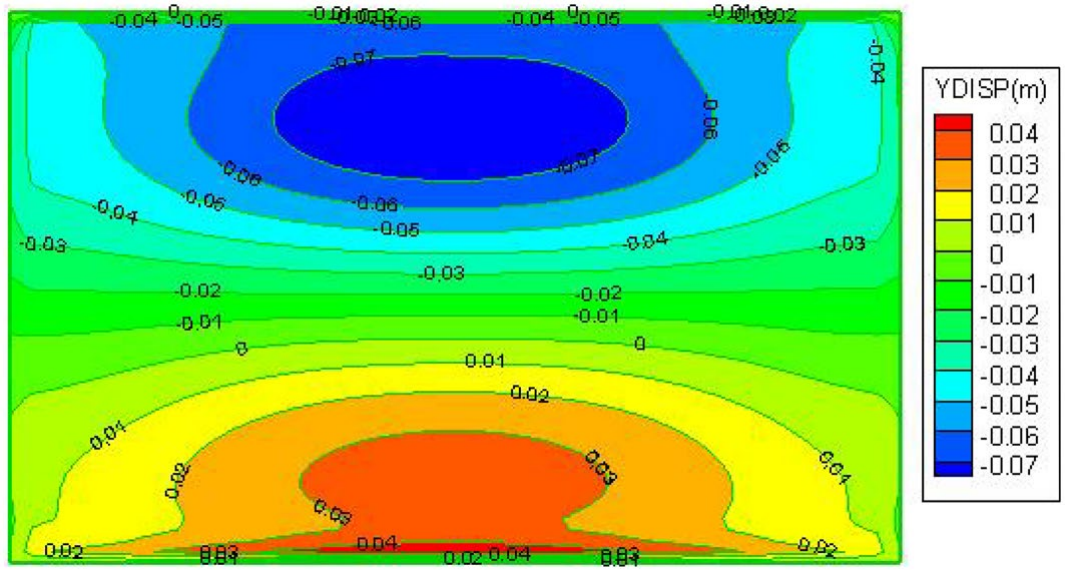
Cloud of horizontal movement of the ground surface along inclination after mining.

**Figure 11:**
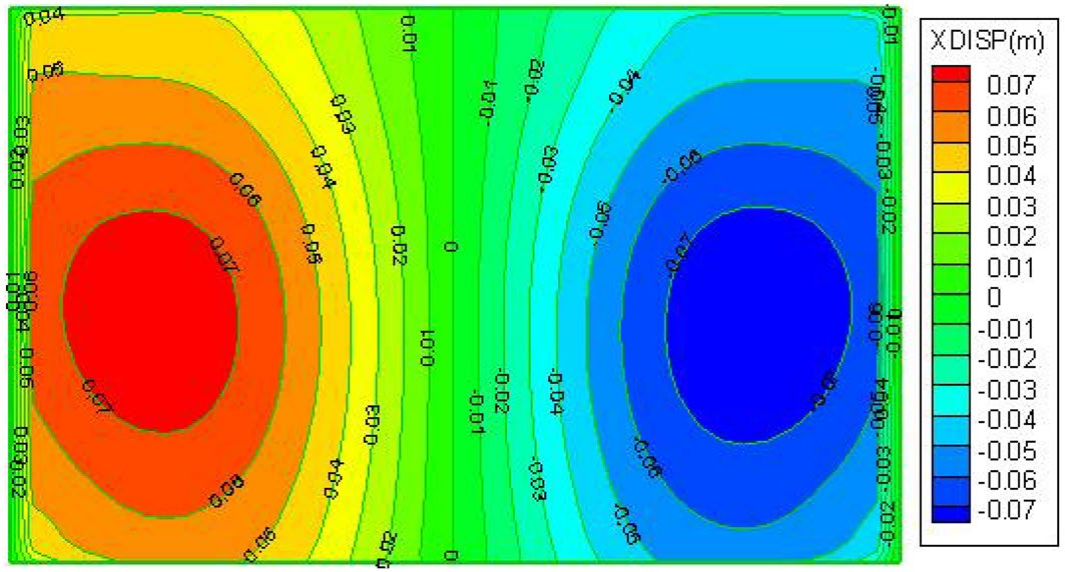
Cloud map of horizontal movement of the ground surface along strike after mining.

The maximum horizontal movement of the surface along the tendency, as depicted in Figs. 10 and 11, is situated between the maximum subsidence value and the boundary of the subsidence basin; the maximum value of horizontal movement is 30 mm in the downhill direction and 80 mm in the uphill direction, the horizontal movement deformation is larger in the up hill direction. Along the trend, the surface's horizontal deformation is more symmetrical, from the subsidence boundary to the subsidence basin, the horizontal stretching deformation gradually decreases, the maximum horizontal stretching deformation is 70 mm.

The results show that after mining the three strips, the maximum surface subsidence was 266 mm, Two maximum horizontal displacements were measured: 80 mm in the Y direction and 70 mm in the X direction, indicating that with the strip mining design of mining a width of 55 m and leaving a width of 75 m, the surface formed a uniform basin and no wave subsidence occurred. Only at the northern boundary of the mining area, the damage to individual buildings reached Class II, which can be repaired and reinforced after mining. In summary, the use of this strip mining design can achieve safe coal mining under the buildings.

**Figure 12:**
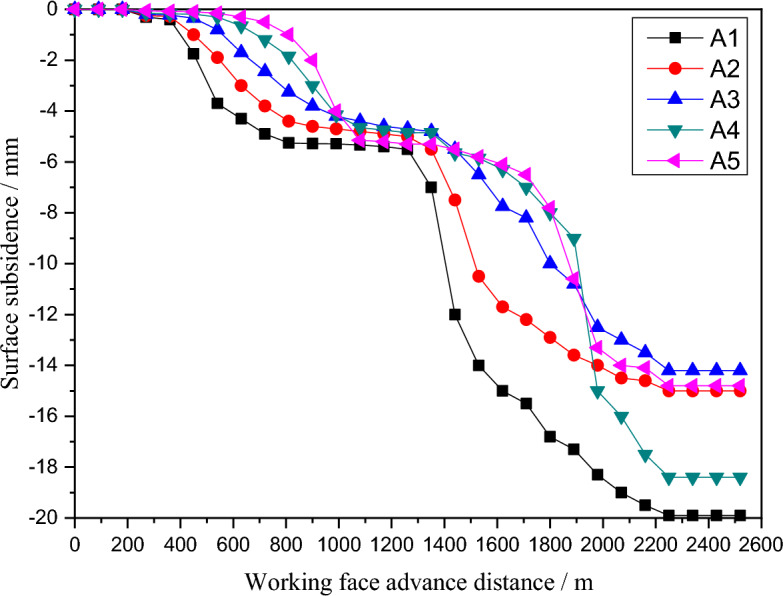
A map of Subsidence curves of A1–A5 measuring point.

**Figure 13:**
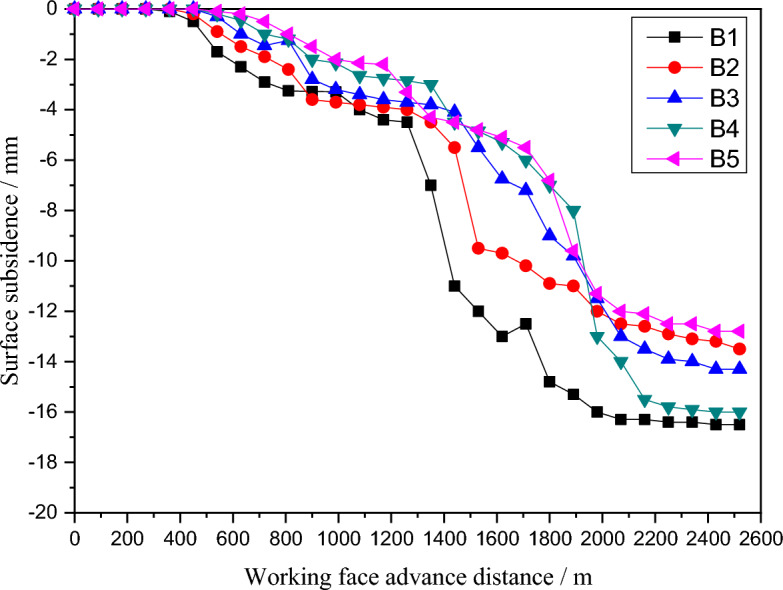
A map of Subsidence curves of B1–B5 measuring point.

In order to further prove the stability of the wide coal pillar and the reasonableness of the design parameters, two observation lines with a total of 10 measurement points, A1-A5 and B1-B5, were laid at the corresponding surface locations in the mining areas of numerical models 21,002, 51,004 and 51,006. The results show that the surface subsidence deformation was relatively slow during the whole mining process, with no sudden change in the value of subsidence, and that the amount of subsidence at each measurement point increased continuously with the mining of the working face, and was only gentle on both sides of the coal pillar in the section, with a maximum subsidence of 160 mm, but the maximum value met the requirements of mining under the building and met the engineering requirements, as shown in Figs. 12 and 13.

In summary, after mining the three strip workings 51,002, 51,004 and 51,006, the maximum surface subsidence value is 275 mm, the maximum horizontal displacement in the Y direction is 80 mm and the maximum horizontal displacement in the X direction is 70 mm, indicating that with the strip mining design of mining width 55 m and leaving a width of 75 m, the surface sinks as a whole after mining without wave subsidence, forming a uniform basin and the surface The damage to buildings was basically controlled within the Class I range. In summary, this strip mining design can be used to achieve safe coal mining under buildings.

## Conclusion

1. The design parameters of strip mining stay width 75 m and mining width 55 m are determined, and pass the verification of strip design coal column stability and recovery rate requirements.
2. After mining, the maximum surface tilt is 1.8 mm/m, the maximum surface subsidence is 210 mm, and the maximum horizontal surface deformation is between 1.0 and − 1.4 mm/m, most of the buildings are damaged less than Class I damage, and only a small part of the three-storey buildings are damaged at Class II level.
3. The FLAC^3D^ numerical simulation calculation show that the strip coal pillars also have sufficient support strength and long-term stability, and the surface sinks as a whole after mining without wave subsidence, forming a uniform basin, and the damage to the surface buildings is basically controlled within the range of Class I.
4. The wide strip mining scheme can maximize the liberation of coal resources under the building, which can safely extract about 1,560,800,000t of coal resources, achieve continuous advance mining of 51,002, 51,004 and 51,006 working faces, reduce moving and overturning of coal mining faces, ensure safe and normal production of the mine, and achieve good economic benefits.

## Data Availability

Part of the data supporting this research result was provided by the company to the author. Still, restrictions apply to the availability of these data, which were used under license for the current study and are not publicly available. Authors will consider reasonable requests for data access from readers and discuss it with data owners for possible permissions. The corresponding author (xakjdxwl@163.com) should be contacted to request the data from this study.
